# Exploiting vulnerabilities of cancer by targeting nuclear receptors of stromal cells in tumor microenvironment

**DOI:** 10.1186/s12943-019-0971-9

**Published:** 2019-03-30

**Authors:** Hong Sheng Cheng, Jeannie Xue Ting Lee, Walter Wahli, Nguan Soon Tan

**Affiliations:** 1 School of Biological Sciences, Nanyang Technological University Singapore, 60 Nanyang Drive, Singapore, 637551 Singapore; 2 Lee Kong Chian School of Medicine, Nanyang Technological University Singapore, 11 Mandalay Road, Singapore, 308232 Singapore; 3 INRA ToxAlim, UMR1331, Chemin de Tournefeuille, Toulouse Cedex 3, France; 4 Center for Integrative Genomics, University of Lausanne, Le Génopode, CH-1015 Lausanne, Switzerland

**Keywords:** Nuclear receptors, Tumor microenvironment, Cancer-associated fibroblast, Myeloid-derived suppressor cells, Tumor-associated macrophage

## Abstract

The tumor microenvironment is a complex and dynamic cellular community comprising the tumor epithelium and various tumor-supporting cells such as immune cells, fibroblasts, immunosuppressive cells, adipose cells, endothelial cells, and pericytes. The interplay between the tumor microenvironment and tumor cells represents a key contributor to immune evasiveness, physiological hardiness and the local and systemic invasiveness of malignant cells. Nuclear receptors are master regulators of physiological processes and are known to play pro−/anti-oncogenic activities in tumor cells. However, the actions of nuclear receptors in tumor-supporting cells have not been widely studied. Given the excellent druggability and extensive regulatory effects of nuclear receptors, understanding their biological functionality in the tumor microenvironment is of utmost importance. Therefore, the present review aims to summarize recent evidence about the roles of nuclear receptors in tumor-supporting cells and their implications for malignant processes such as tumor proliferation, evasion of immune surveillance, angiogenesis, chemotherapeutic resistance, and metastasis. Based on findings derived mostly from cell culture studies and a few in vivo animal cancer models, the functions of VDR, PPARs, AR, ER and GR in tumor-supporting cells are relatively well-characterized. Evidence for other receptors, such as RARβ, RORγ, and FXR, is limited yet promising. Hence, the nuclear receptor signature in the tumor microenvironment may harbor prognostic value. The clinical prospects of a tumor microenvironment-oriented cancer therapy exploiting the nuclear receptors in different tumor-supporting cells are also encouraging. The major challenge, however, lies in the ability to develop a highly specific drug delivery system to facilitate precision medicine in cancer therapy.

## Background

In human cells, there are 48 nuclear receptors (NRs) that play integral roles in numerous physiological functions such as metabolism, cell development, immunity, and stress response. Classically, following direct lipophilic ligand binding, NRs will recognize and bind to specific DNA motifs across the genome, which are known as NR response elements. The binding of an NR to its response element and transcriptional activation of target genes often require homodimerization of NRs or heterodimerization with retinoid X receptor (RXR) coupled to the recruitment of coactivator proteins, although certain receptors are functionally active as a monomer [1, 2]. Independent of ligand binding, the activities of NRs can also be modulated by posttranslational modifications such as phosphorylation, ubiquitination, and SUMOylation, or indirect recruitment to the genome by other DNA-bound transcription factors via tethering mechanisms [2, 3]. Increasing evidence has also unveiled the pivotal roles of NRs in chromatin remodeling [4]. Furthermore, certain NRs such as progesterone receptor (PR) and peroxisome proliferator-activated receptor (PPAR)-γ possess different isoforms resulting from alternative splicing. Variations in the tissue expression profile, ligand affinity and target genes between different isoforms have been reported, further enlarging the scope of the cellular events coordinated by NRs [5, 6] Hence, given the complex and multifaceted regulatory network coordinated by NRs, their impacts on human physiology are undoubtedly highly consequential.

In drug development, NRs are ideal therapeutic targets because their activities can be readily induced or repressed with small molecules that mimic their natural ligands, allowing fine manipulation of the biological functions or pathological processes controlled by the receptors. This possibility is particularly true for endocrine receptors such as thyroid hormone receptor (THR), vitamin D receptor (VDR), estrogen receptor (ER), androgen receptor (AR), glucocorticoid receptor (GR) and PR, as well as adopted orphan receptors such as farnesoid X receptor (FXR), RAR-related orphan receptor (ROR) and PPARs with well-characterized endogenous ligands. In this context, the involvement of NRs in various types of cancer has been extensively documented [7, 8]. Clinically, strategies that aim to block AR and ER, namely, androgen deprivation therapy and selective ER modulators, are widely employed to treat prostate and breast cancer, respectively, strongly supporting the practicality of NRs as druggable targets to improve cancer treatment outcomes.

Recently, the tumor microenvironment (TME) has swiftly garnered the attention of the cancer research community and has been accepted as the key contributor to tumor progression. The interplay between TME and the tumor epithelium empowers the aggressiveness of tumor cells by enhancing tumor proliferation, chemoresistance, immune evasion and metastatic tendency [9]. Other than cancer cells, TME is populated by highly heterogeneous groups of cells, including cancer-associated fibroblasts (CAFs), tumor-associated macrophages (TAMs), endothelial cells, adipose cells, myeloid-derived suppressor cells (MDSCs), and other immune and inflammatory cells. All members of the microenvironment function cooperatively with the assistance of a vast variety of cytokines, chemokines, growth factors, and other signaling molecules, to compose a dynamic and ever-evolving network that offers sharpened stress responses and enhanced survivability to the malignant cells [9].

**Fig. 1:**
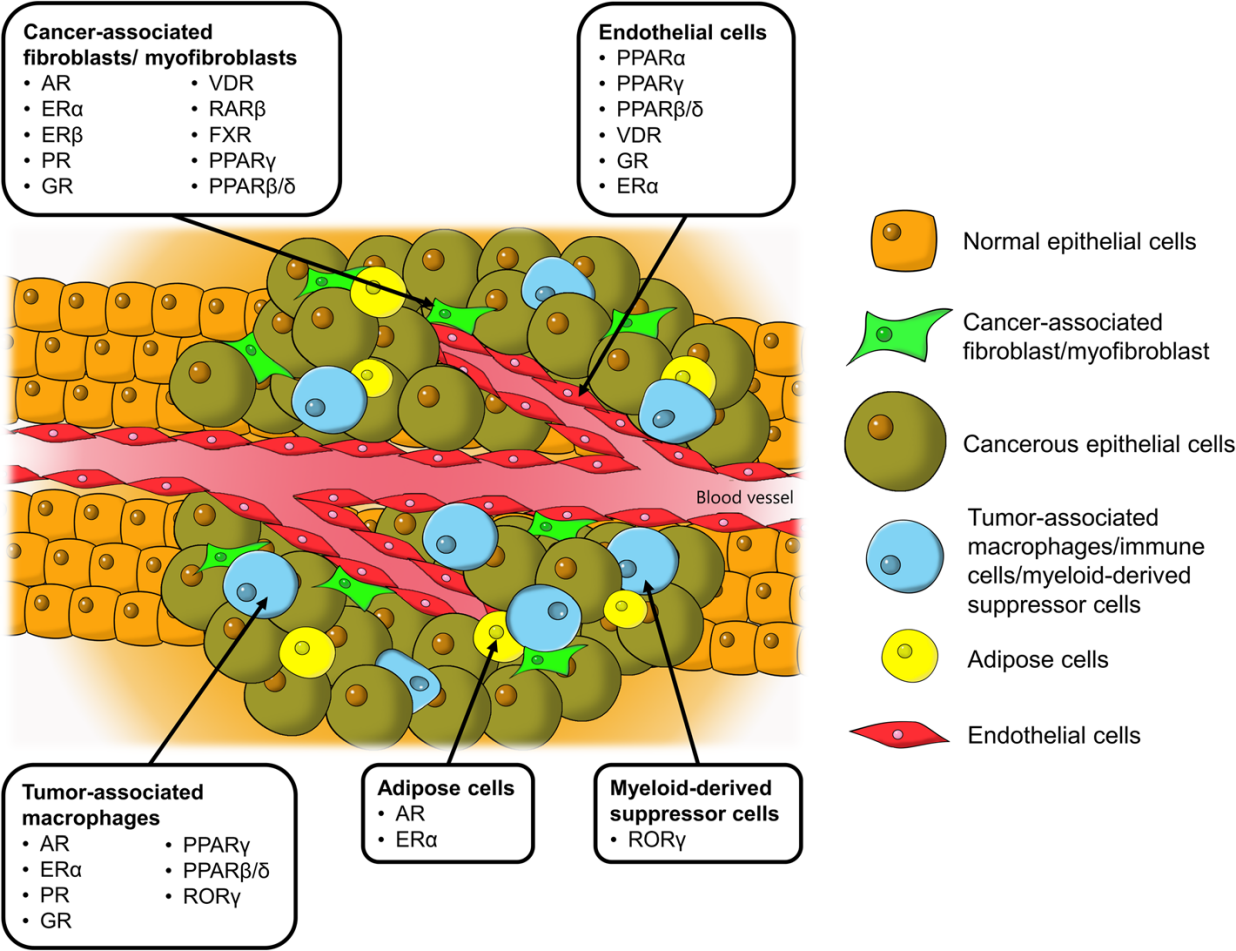
Tumor microenvironment, tumor-supporting cells and the identified nuclear receptors in cancer progression. AR, androgen receptor; ER, estrogen receptor; FXR, farnesoid X receptor; GR, glucocorticoid receptor; PPAR, peroxisome proliferator-activated receptor; PR, progesterone receptor; ROR, RAR-related orphan receptor; VDR, vitamin D receptor

In this context, although NRs in tumor cells have been widely studied, their implications in TME are comparatively underappreciated. Given the pro-oncogenic roles of TME as well as the pronounced regulatory effects and excellent druggability of NRs, understanding the roles of these receptors in TME is of great interest. The implicated NRs in various tumor-supporting cells in TME presented in this review are illustrated in Fig. 1. Knowledge of the NR expression profile not only helps to provide a fundamental understanding in the realm of cancer biology but also harbors enormous clinical value in cancer therapy. Thus, this review aims to highlight key findings of the biological functions of NRs in different cell types presented in TME in relation to their pro−/ anti-tumor activities. The empirical findings are also discussed concerning the challenges, limitations and future direction of the current research paradigm with high hopes of developing a new anti-cancer strategy by exploiting NRs in TME.

### Cancer-associated fibroblasts/myofibroblasts as key accomplices in tumor malignancy

#### Regulatory roles of CAF steroid hormone nuclear receptors in hormone-dependent cancers

Forming one of the most abundant cell populations in TME, CAFs are known to be pivotal modulators of tumorigenicity and cancer progression. A much larger number of studies have been conducted on CAFs than on other stromal cells in TME, particularly in terms of steroid hormone NRs. Therefore, this review of the actions of NRs in CAFs is subdivided into two parts in accordance with steroid and nonsteroid hormone NRs.

CAFs are primarily composed of fibroblasts and myofibroblasts, of which the latter displays a mixed phenotype of fibroblast and smooth muscle cells by having a prominent rough endoplasmic reticulum of fibroblasts and contractile filaments (e.g.*,* smooth-muscle actin) of smooth muscle cells [10]. The crosstalk between the tumor and CAFs assists tumor cells in acquiring unique characteristics such as enhanced proliferation, metastatic and angiogenic properties, immune evasion and chemoresistance [11, 12]. It has been postulated that dysregulated activities of certain nuclear factors in CAFs could contribute to their tumor-supportive roles. CAFs have markedly distinct gene expression profiles of NRs compared with their normal cognate fibroblasts. Indeed, CAFs isolated from human breast tumors exhibit vastly different NR fingerprints compared with normal breast fibroblasts, as exemplified by the downregulation of THR-β, VDR, ROR-α, and PPAR-γ in CAFs [13]. Furthermore, NR signatures also differ among CAFs isolated from different types of tumors [13–15]. Such disparities in NR profiles could be an intrinsic characteristic of fibroblasts at different anatomical positions, or due to cellular signals released by different host cancer cells and other surrounding stromal cells. In this context, our recent study using clinical cutaneous squamous cell carcinoma has confirmed the differential gene expression of NRs in CAFs compared with normal fibroblasts [15]. We have also shown that the transcriptomes of tumor cells cocultured with CAFs can be altered by reversing the expression pattern of selected NRs, namely, PPARβ/δ, VDR, AR and retinoic acid receptor (RAR)-β receptor, to result in functional changes such as impaired invasiveness, reduced proliferation, and altered energy metabolism and redox response [15]. More importantly, when the squamous cell carcinoma cultures are exposed to conditioned medium from CAFs pretreated with either RARβ or AR antagonists, the CAF-induced cisplatin resistance is completely abolished [15]. Our study strongly supports the druggability of NRs in TME, notably AR and RARβ, which can mediate a CAF-directed cancer therapy.

In line with our findings, AR in the tumor stroma has been consistently found to be a predominant factor in the prognosis of prostate cancer [16]. Nevertheless, unlike squamous cell carcinoma, in which the inhibition of AR of CAFs could be beneficial, low levels or loss of AR in the stromal cells of prostate cancer are associated with poorer clinical outcomes [17–22]. Such an association is mind-boggling given that androgen deprivation therapy, which aims to suppress AR signaling in tumor cells, often serves as the frontline treatment of prostate cancer [23]. Genome-wide CHIPseq has revealed that AR in prostate CAFs has distinct binding sites and binding sequence motifs compared with tumor cells, suggesting differences in AR-regulated genes between the two cell populations [24]. This finding could explain the discrepancy in AR function between prostate CAFs and cancer cells. The tumor stroma liberates various androgen-responsive growth factors and cytokines that modulate the cell fate, proliferation and drug sensitivity of prostate cancer cells [25–27]. These paracrine factors are favorable for the growth of tumor cells present in this environment. Although ablation of ARs in CAFs could attenuate cancer proliferation [28], the loss of AR signaling activity is also linked to the onset of metastatic phenotypes such as increased stemness, enhanced cell migration and weakening of the extracellular matrix (ECM) structure and integrity [22, 29, 30]. As a result, the suppression of AR in CAFs may potentially exacerbate the epithelial-mesenchymal transition and metastasis of prostate cancer, underpinning the association of AR loss in CAFs with adverse clinical outcomes in prostate cancer progression. In short, the pathological roles of AR in CAFs are well-implicated in the development of prostate cancer, making it an attractive therapeutic target. However, considering the opposite effects of AR blockade in tumor and stromal cells, an ideal anti-androgenic agent should decrease tumor AR but enhance stromal AR activity [16]. It is also worth mentioning that the current understanding of AR in CAFs is mostly derived from hormone-dependent tumors, especially prostate and breast cancers [16, 31]. Thus, in light of the evidence mentioned above, it is worthwhile to extend research on AR to other types of tumors to better characterize its roles in cancer biology.

In addition to AR, steroid hormone NRs in CAFs, including ERα and β, PR and GR, are also relatively well-studied. The expression of ERα has been detected in the CAFs of breast [13], endometrial [32], cervical [33] and prostate cancers [34], but not in colorectal carcinoma [35]. However, the clinical implications of ERα are diverse. In some studies, ERα-expressing CAFs have been reported to promote prostate and endometrial cancer cell proliferation [32, 36]; in other studies, CAFs attenuated prostate tumor cell invasiveness and immune cell infiltration by altering the levels of anti-angiogenic factors, ECM remodeling factors as well as chemokines, in addition to conserving chemosensitivity in certain breast cancer cell lines [37–39]. Similarly, divergent results have also been obtained in clinical biopsies, in which one association study found a positive correlation between ERα expression in CAFs with advanced prostate cancer stage [34], while the reverse trend was found in cervical cancer [33]. Despite these perplexing findings, a recent comparative transcriptomic study demonstrated differential expression patterns between CAFs isolated from early- and late-stage cervical cancer, with the latter being more metabolically and proliferatively active upon estradiol exposure [40]. Treatment with ER antagonists, namely, ICI182780 and methylpiperidino pyrazole, not only reverses the aforementioned changes but also suppresses the expression of genes linked to angiogenesis and cell adhesion [40]. Additionally, liver receptor homolog-1 (*LRH-1*), which is an orphan NR, is transcriptionally responsive to estrogen treatment and ERα activation [41]. In breast cancer-derived CAFs, LRH-1, which is highly expressed in these cells, can upregulate aromatase (*CYP19*) gene expression [13, 42]. This observation is indicative of an ERα-mediated loop of estrogen biosynthesis via LRH-1 in CAFs, which may contribute to the increased tumor cell proliferation. Hence, disrupting the paracrine signaling directed by ERα in CAFs may be beneficial, making NR an exploitable target for cancer therapy. However, further investigation is warranted to clarify the conflicting results about the tumorigenic properties of ERα.

While ERα is well-implicated in TME of many hormone-dependent cancers, its role is less pronounced in the CAFs of breast cancer, likely because its expression is predominantly localized in the tumor epithelium instead of the surrounding fibroblasts [43–45]. In contrast, ERβ, which is the other ER isotype, is widely found in the breast cancer stroma [35, 46]. Despite their structural similarities, the bioactivities of ERα and β in tumor epithelium are largely counteractive, whereby ERβ is anti-proliferative and ERα-antagonizing [47, 48]. Whether ERβ in CAFs also confers an anti-tumor effect is uncertain. One study revealed that progesterone and epidermal growth factor receptors are highly expressed in the uterine stroma of ERβ-knockout mice, especially when 17β-estradiol and progesterone are coadministered [49]. This phenomenon contributed to the hyperproliferation and impaired cellular differentiation observed in the uterine epithelium of ERβ-knockout mice [49]. Conversely, PR also exhibits ERα-antagonizing properties in tumor cells [50]. Its expression in cancer-associated stroma is repressed in comparison to benign stroma in prostate glands [51, 52]. Stromal PR actively takes part in stromal cell differentiation [52]. Although conditioned medium from PR-positive CAFs has a negligible effect on prostate cancer cell proliferation, cell motility and migration are vastly inhibited via the suppression of stromal-derived factor-1 and interleukin (IL)-6 [51]. These findings highlight the importance of stromal ERβ and PR in stroma-tumor epithelium crosstalk in modulating cancer progression, but tissue-specific inhibition or activation of these NRs in CAFs is imperative to outline the feasibility of exploiting them as therapeutic cancer targets.

Next, GR is differentially expressed in TME compared with normal tissues [53], with remarkably high expression in CAFs [54, 55]. In cancer-associated myofibroblasts, treatment with dexamethasone successfully induces nuclear translocation of GR, resulting in an anti-inflammatory phenotype marked by the repression of IL-1β, monocyte chemoattractant protein 1, C-C motif ligand 5, tumor necrosis factor-α (TNFα) and intercellular adhesion molecules [56]. Coincidentally, several pro-invasive paracrine signals, such as tenascin C, hepatocyte growth factor, transforming growth factor β (TGFβ), are also significantly suppressed [56]. Further investigation showed that dexamethasone-induced activation of GR in myofibroblasts, but not in cancer cells, can nullify the proliferative effect of myofibroblasts on tumor cells and potentially inhibit epithelial-mesenchymal transition, but it is associated with pro-migratory behavior [57]. Apart from the tumor epithelium, paracrine factors from myofibroblasts also interact with the surrounding endothelial cells to promote cell motility and angiogenesis [58]. These activities are dampened by the conditioned medium from dexamethasone-treated myofibroblasts together with a decline in urokinase-type plasminogen activator and angiopoietin-like protein-2 [58]. In general, GR activation in myofibroblasts exhibits tumor-inhibiting effects. It is, however, noteworthy that current evidence for this phenomenon originated from one research group, rendering further validation pertinent.

#### Nonsteroid hormone nuclear receptors - Anti-tumor properties of VDR, PPARγ, RXR and FXR and pro-tumor effects of PPARβ/δ and RARβ in CAF

In addition to steroid hormone NRs, VDR in CAFs is also increasingly appreciated as a key anti-carcinogenic target. Ferrer-Mayorga et al. (2017) reported a positive correlation between the gene expression of stromal VDR with overall survival and progression-free survival in colorectal cancer [59]. Genes such as *CD82* and *S100A4*, which are responsive to calcitriol in CAFs, are also associated with clinical outcomes and stromal VDR expression in patients with colorectal cancer, supporting a clinical value of VDR agonists in cancer treatment [59]. Conversely, pancreatic and hepatic TME is enriched by myofibroblast-like stellate cells, which upon activation, become proinflammatory, fibrogenic and tumor supportive [60, 61]. Based on a transcriptomic analysis, calcipotriol, which is a nonhypercalcemic vitamin D analog, maintains the quiescent state and modifies the secretomes of pancreatic stellate cells by reducing the expression of inflammatory cytokines, ECM components, and growth factors [62]. Similar trends have also been observed in hepatic stellate cells [63, 64]. Combined therapy with gemcitabine plus calcipotriol tremendously improves the treatment outcomes of mice with orthotopic pancreatic ductal adenocarcinoma transplant, as evidenced by intratumoral aggregation of chemotherapy agents, a diminished tumor size and a higher survival rate [62]. A very recent report also suggests a regulatory role of VDR on CAF-liberated exosomal miRNA (e.g., miR-10a-5p and miR-181a-5p) [65]. Hence, exposure of CAFs to VDR ligands may modulate the stroma-tumor crosstalk not only via paracrine signaling but also by manipulation of the exosomal content. Despite promising results from preclinical studies, most clinical trials that employed vitamin D for cancer therapy and prevention have yielded underwhelming results, which reflects an inadequate understanding of VDR actions in both tumor and stromal cells [66–68]. Thus, an in-depth dissection of the biological roles of VDR in TME is critical to enable effective VDR-centric cancer treatment.

Several studies have also examined the activities of PPARs in CAFs. PPARγ has been found to be highly expressed in the myofibroblasts of colon adenocarcinoma biopsies, but not in normal colon tissues [69]. When hypoxic breast tumor cells are exposed to pioglitazone (PPARγ agonist) and/or 6-OH-11-O-hydrophenanthrene (RXR agonist), the resultant exosomes are unable to trigger CAF activation compared with exosomes from tumor cells subjected to the control treatment, suggesting that these NR agonists can disrupt the tumor-stroma crosstalk [70]. In the same study, coactivation of PPARγ and RXR in CAFs was found to effectively silence the pro-inflammatory response and metastatic phenotype by suppressing the expression of IL-6, carbonic anhydrase IX, metalloproteinase (MMP)-2 and MMP9 [70]. A similar anti-proliferative effect of PPARγ activation on melanoma-derived CAFs has also been reported using the PPARγ agonist 15d-PGJ2 [71]. Accordingly, activation of PPARγ in CAFs could potentially act as a tumor suppressor by modifying the activation and supportive properties of CAFs in cancer development. Unlike PPARγ, which is associated with anti-tumor effects upon ligand binding, PPARβ/δ in CAFs has a pro-tumor action. This phenomenon was clearly demonstrated in our recent study, in which the tumor burden was significantly lowered in fibroblast-specific PPARβ/δ knockout mice subjected to either chemical (azoxymethane or dextran sulfate sodium), genetic (APC^min/+^) or combinatory (APC^min/+^ with dextran sulfate sodium) tumorigenic induction [72]. Mechanistically, PPARβ/δ ablation in CAFs significantly escalates H_2_O_2_ liberation into the TME, exposing the tumor epithelium to increased oxidative stress to subsequently trigger NRF2-mediated signaling that attenuates tumor growth [72]. The regulatory effects of PPARβ/δ on oxidative stress, reactive oxygen species production, and antioxidant mechanism are in line with a previous study examining the wound microenvironment [73]. In short, both PPARγ and PPARβ/δ in CAFs play a significant modulatory role in cancer development, of which the former acts on the local inflammation and cancer invasiveness while the latter alters the redox balance in TME.

FXR is an integral regulator of genes responsible for lipid, cholesterol and bile acid metabolism [74]. Loss of function of FXR is strongly linked to carcinogenesis in the liver, intestines and colorectal region where the receptor is highly expressed [75, 76]. Interestingly, in breast cancer cells exposed to the FXR agonist GW4064, conditioned medium from CAFs fail to promote enhanced growth, motility, and invasiveness [77]. This observation reflects a neutralizing effect of FXR activation on the tumorigenic paracrine signaling conferred by CAFs. Likewise, the characteristics of CAFs subjected to GW4064 are also profoundly altered. For instance, the genes involved in the cytoskeleton and cellular movement as well as a wide variety of growth factors are significantly downregulated, subsequently leading to loss of the tumor-supportive effects of CAFs [78]. The ability of an FXR inhibitor, guggulsterone, to completely reverse the GW4064-mediated anti-tumor effects further corroborates the necessity for FXR activation in eradicating the tumor-promoting features of CAFs [68]. In short, the evidence thus far for the benefits of FXR activation in CAFs is scarce, yet remarkably promising [78].

As mentioned earlier, our group has demonstrated that suppression of RARβ in CAFs via genetic knockdown or with an antagonist named LE135 consistently lowers the chemoresistance of tumor cells that are otherwise promoted by wild-type/untreated CAFs [15]. This result also complements a previous study that concluded that RARβ inhibition creates a hostile microenvironment that suppresses tumorigenesis through stromal remodeling, including impaired angiogenesis and reduced inflammatory cell recruitment and cancer-associated myofibroblast numbers [79]. In fact, our study also predicts that activation of VDR and GR, as well as inhibition of AR in CAFs, can potentiate the efficacy of chemotherapy, all of which are in excellent agreement with current understanding of these NRs in CAFs, as discussed previously. Collectively, based on preliminary data from various sources, NRs in CAFs or myofibroblasts are undoubtedly druggable targets that could serve as a new strategy to improve the clinical outcomes of pre-existing therapeutic approaches. For certain receptors such as AR and ERα, their pro-oncogenic roles in CAF could be dependent on the cancer types and biochemical signals, resulting in the contradictory findings obtained thus far. Hence, diversifying the research to other cancer types and escalating cell-based methodology to preclinical animal study are commendable efforts to strengthen the concept and clinical prospects of CAF-oriented cancer therapy via NR inhibition.

### The steroid hormone nuclear receptors PPARs and RORγ are crucial mediators of TAM and MDSC formation

Apart from CAFs, TME is also occupied by numerous bone marrow-derived cells such as TAMs, MDSCs, neutrophils and tumor-infiltrating lymphocytes. Among these cells, TAMs and MDSCs are known to exhibit evident tumor-supporting and immune suppressive activities [80, 81]. Like CAFs, the steroid hormone NRs in TAMs also have profound impacts on cancer progression. It is widely accepted that TAMs, which more closely resemble alternatively activated M2 macrophages, are activated by Th2 cytokines such as IL-4, IL-10, and IL-13 [82]. M2 macrophage polarization is also promoted by exposure of the monocytes to glucocorticoids, which stimulates GR activation [83]. This process is accompanied by a significant downregulation of proteins linked to lysosomal activity, antigen presentation, and proinflammatory proteins, indicating immunosuppressive effects [83]. Additionally, GR also functions synergistically with p38MAPK to regulate the expression a CD20 homolog, MS4A8A, the overexpression of which in TAMs significantly enhances the tumor burden [84]. Taken together, classic GR signaling may play a dominant role in the tumor-supporting activities of TAMs.

In contrast to GR, the role of AR, ER, and PR-dependent tumorigenesis is poorly defined. The presence of TAMs influences the expression of ERα, ERβ and PR in tumor cells [85–87]. Reciprocally, the number of TAMs also appears to be modulated by steroid hormone NRs of tumor cells, particularly ER [88]. Moreover, in wound healing and lung inflammatory studies, activation of AR, ERα and PR by their cognate steroid hormones would favor macrophage activation in an alternative manner, producing M2 macrophages that compel cellular repair and angiogenic processes [89–91]. The studies suggest that steroid hormones are vital determinants in the alternative differentiation of macrophages to modulate pulmonary inflammation and wound recovery. However, there is no direct evidence supporting the contribution of AR, ER, and PR to the formation of M2 macrophages in TME. Thus, future research should focus on explicating the roles of these NRs in TAM formation and tumor-supporting events.

The three isotypes of PPARs, PPARα, PPARβ/δ, and PPARγ, are widely known to influence carcinogenic activities. However, current evidence is somewhat paradoxical concerning their roles in tumor cells, leading to the speculation that their actual functions could be dependent on the ligands, cancer types or even cancer stages [92]. In immune cells, PPARs also govern the fate of macrophage activation, likely because the maturation of macrophages is tightly linked to their metabolic state. To enable alternative activation of macrophages, immune cells must undergo oxidative metabolism, which is modulated by PPARs [93]. Macrophages that are unable to clear the metabolic checkpoint due to deletion of PPARγ, PPARβ/δ and PPARγ coactivator 1β (PGC-1β), are incapable of expressing the alternative phenotype [94–96]. In contrast, treatment with PPARα or -γ agonists fosters the enrichment of M2-related biomarkers in macrophages [97]. Recently, a ligand-independent mechanism that involves PPARγ in TAM differentiation has also been described, which involves the cleavage of PPARγ by caspase-1 and thereby produces a 41-kDa receptor fragment that translocates into mitochondria and interacts with medium-chain acyl-CoA dehydrogenase [98]. This interaction shuts down the enzyme and attenuates fatty acid oxidation, leading to intracellular aggregation of lipid droplets that drive TAM differentiation [98]. These results support the pro-tumor activities of PPARγ via promoting TAM formation. Likewise, PPARβ/δ also seems to follow a similar trajectory [99]. Notwithstanding, other empirical findings support a counterargument [100, 101]. The clinical use of thiazolidinedione is also not associated with an increased risk of many malignancies [102]. Collectively, the roles of PPARs in TAM differentiation and tumor progression undoubtedly remain an open topic necessitating further investigation.

RORs are classified as orphan NRs, which belong to a subfamily of thyroid hormone-like receptors. RORs are subcategorized into RORα, −β and -γ, the last of which is highly expressed in thymus and lymphoid tissues and linked to immune cell differentiation and immune system regulation [103]. Interestingly, RORγ is also a crucial element in hematological malignancies. For example, RORγ knockout mice are predisposed to thymic and lymphoblastic lymphomas [104, 105]. In addition, patients with multiple myeloma display an overexpression of RORγ in their peripheral blood mononuclear cells [106]. The roles of RORs in tumorigenesis vary in different cancers [103]. Nonetheless, in TME, activation of RORγ with an agonist (SR1078) promotes the formation of MDSCs and TAMs [107]. RORγ-dependent myelopoiesis is mediated by key regulators such as Socs3, Bcl3, and C/EBPβ, as well as macrophage-specific transcription factors, including IRF8 and PU.1 [107]. In the same study, RORγ could confer pro-tumor effects by shielding MDSCs from apoptotic death, promoting tumor growth and restricting tumor-infiltrating neutrophils, while ablation of the receptor successfully attenuates these processes [107]. These results position RORγ as an attractive target, and hence, the pharmacological effects of RORγ antagonists or inverse agonists in TAMs and MDSCs with respect to tumor development are of immense interest.

To summarize, research on NRs in TAMs or MDSCs is still in its infancy. Most of the available studies emphasize the effects of NRs on the fate of macrophage activation. This information is critical not only to inhibit the alternatively activated M2 macrophage pathway, which subsequently reduces the TAM count, but also to achieve reprogramming of M2 to M1 macrophages to initiate tumoricidal effects such as the induction of proinflammatory and anti-tumor immune responses in TME.

### Ceasing angiogenesis - targeting GR, PPAR and VDR of endothelial cells in TME

The vascular endothelium is an essential tissue that maintains blood perfusion in addition to regulating the trafficking of nutrients and leukocytes to surrounding tissues. In TME, the integrity of the vascular endothelium is often jeopardized by factors such as hypoxia and chronic growth factor stimulation. Genetic abnormalities are also not uncommon in tumor endothelial cells [108]. As a cumulative result of atypical physiological conditions and genetic mutations, tumor endothelial cells differ significantly from normal endothelial cells by being highly proliferative, pro-angiogenic and more disorganized and leaky regarding the vasculature [109, 110].

Recent cancer research has identified PPARs as potential therapeutic targets and prognostic indicators for cancer therapy. Indeed, the expression of PPARγ is associated with slower progression and a lower incidence of tumor recurrence in bladder cancer [111]. This correlation is lost when certain angiogenic factors, namely, basic fibroblast growth factor and platelet-derived endothelial growth factor, are coexpressed in the tumors, indicating a possible role of PPARγ in angiogenesis in cancer progression by interacting with these growth factors [111]. Activation of PPARγ in endothelial cells is predominantly linked to anti-angiogenic activities, as exemplified by decreased expression of pro-angiogenic factors, reduced proliferation, impaired endothelial cell migration and tubule formation [112], but conflicting results have also been reported [113, 114]. Similar to PPARγ, fenofibrate-induced PPARα activation in various tumor cell lines concomitantly suppresses proangiogenic vascular endothelial growth factor (VEGF) biosynthesis and increases anti-angiogenic thrombospondin 1 and endostatin [115]. These bioactivities are translated into reduced endothelial cell proliferation and neovascularization as well as impaired growth of the subcutaneous tumor xenograft in mice [115]. Unlike PPARα and –γ, PPARβ/δ appears to be proangiogenic. Treatment with the PPARβ/δ ligand GW501516 promotes endothelial tube formation, whereas the maturation of microvessels in tumors is severely disrupted in PPARβ/δ knockout mice, leading to diminished blood flow to the tumors [116, 117]. Taken together, all three isotypes of PPARs are actively involved in the angiogenesis performed by endothelial cells, which is one of the most critical processes in cancer development, sustaining the rapid expansion of tumor cells and opening the window for the metastatic process. However, the findings are not strictly based on tumor-derived endothelial cells. Given the functional variations between tumor-associated and normal endothelial cells, further validation is pertinent.

Next, VDR is closely associated with the development of endothelial cells in TME. In this context, calcitriol, which is an active metabolite of vitamin D, has been widely studied regarding its roles in bone and mineral metabolism, as well as the differentiation of both normal and malignant cells. At a low dosage, calcitriol exhibits an anti-proliferative effect on cancer cells such as breast, colon, and prostrate while promoting differentiation, cell cycle arrest and eventually apoptosis [118]. A similar growth inhibitory effect has also been observed in tumor-derived endothelial cells, but not in normal ones [119]. Generally, increased levels of VDR ligands trigger a self-regulatory pathway by enhancing the expression of CYP-24b, a key enzyme in vitamin D catabolism [120]. As a result, VDR ligands are degraded and unable to trigger VDR-mediated anti-proliferative effects [121]. However, overexpression of CYP-24 has been reported in various cancers such as prostate, colon and breast cancer, explaining the varying calcitriol sensitivity and calcitriol resistance in these patients [122]. Moreover, the anti-proliferative effect of VDR in endothelial cells also relies on the epigenetic silencing of *CYP-24,* which is achieved via hypermethylation at the CpG islands of *CYP-24* promoter regions [123]. Transcriptional activation of *CYP-24* is prevented by the hypermethylation pattern, leading to growth inhibition in tumor-derived endothelial cells [123]. One study has also suggested a link between VDR and angiogenesis in TME modulated by a pro-oncogenic protein named DKK-4 [124]. The expression of DKK-4 is inversely correlated to that of VDR, while endothelial cells are more prone to migrate and form microvessels when they are exposed to conditioned medium from DKK-4-expressing cells [124]. The pro-tumor effects of DKK-4 are effectively eliminated by treatment with calcitriol. Thus, these studies support the use of VDR ligands that target the tumor endothelium with minimal disturbance to the normal vasculature.

Multiple studies have demonstrated the anti-angiogenic effects of glucocorticoids in normal and malignant cells, as well as during wound healing [125, 126]. In tumor cells, glucocorticoids exert a direct inhibitory effect on the secretion of VEGF, which can be reversed by GR antagonist treatment [127]. This observation suggests that the anti-angiogenic effect is GR-dependent. Logie et al. (2010) reported that glucocorticoids have a negligible effect on the proliferation, viability and migration properties of endothelial cells, but instead, the hormone enhances thrombospondin-1 expression and impairs cell-cell contact, thus preventing the formation of endothelial tubules even in the presence of VEGF and prostaglandin F_2a_ [128]. The potent angiogenic inhibitory activity of GR has also prompted research on the nanosized drug delivery system to maximize the anti-tumor effect of GR [129].

Unlike GR, ERα is linked to the pro-angiogenic process in TME. Treatment with 17β-estradiol increases the vessel density and stabilizes the endothelium vasculature in tumors, making the blood vessels more resistant to insults from hypoxia and necrosis [130]. Increased neovascularization in the tumor environment ensures adequate oxygenation of the tumors and minimizes tumor cell death due to the hypoxic environment [130]. However, ERα-dependent angiogenesis is primarily mediated by Tie2-expressing cells, which are not of hematopoietic origin [130]. Therefore, the true identity of Tie-2 positive cells in TME, and their relationship with tumor endothelial cells, remain to be clarified.

### Adipose cells are emerging players in tumor aggressiveness

Adipocytes, also known as fat cells, are regulators of human physiological processes such as tissue homeostasis, and they are the primary site for energy storage in the form of intracellular triglycerides packaged in lipid droplets [131]. Additionally, they are also endocrine cells that secrete hormones and cytokines to regulate human physiological processes such as inflammation and the reproductive system [132]. The functions of adipose cells in TME resemble those of fat depots, but in a tumor-supportive manner [133]. Emerging evidence also supports a role for dysfunctional adipose tissues in field cancerization mediated by prolonged local inflammation [134]. However, our understanding of the role of adipose cells in TME is still considerably limited.

One recent study has shown that the recruitment of preadipocytes occurs more readily in prostate cancer cells than normal prostate tissues, a process that enhances the invasiveness of prostate cancer in mice with orthotopic xenografts [135]. Mechanistically, neighboring adipocytes significantly increase the expression of miRNA-301a in tumor cells, which serves to suppress AR signaling in these cells [135]. The inhibition of AR signaling is followed by alterations in the gene expression of TGF-β via the serine/threonine kinase receptor or TGF-β receptor and its downstream genes such as Smad3 and matrix-metalloproteinase-9, fueling metastatic processes [135]. Coculturing human Simpson Golabi Behmel Syndrome (SGBS) preadipocyte cells, which are considered to be a representative in vitro model of white preadipocytes, and ER-positive MCF7 breast cancer cells results in the suppression of ERα expression in MCF7 cells [136]. Cohabitation of preadipocytes and MCF7 cells also significantly enhances the epithelial-mesenchymal transition of MCF7 tumor cells, as documented by overexpression of FOXC2 and TWIST1, and changes in N- and E-cadherin expression [136]. As a consequence, the expression of HIFα, TGF-β and lectin-type oxidized LDL receptor 1 in SGBS adipocytes are elevated [136]. Both studies have demonstrated that the presence of adipose cells in TME can impact both NR signaling and oncogenic processes in cancer cells. However, the studies did not aim to delineate the activities of NRs in tumor-associated adipose cells and their contribution to cancer progression, an aspect that has been minimally explored to date. In light of the emerging roles of adipose cells in field cancerization as well as the predominant actions of various NRs in adipocyte biology, it will be interesting to unearth this relationship.

### Implications of existing research for stroma-directed anticancer therapy via nuclear receptor manipulation

For years, targeting the tumor epithelium has been the sole cornerstone of cancer research, which has resulted in the clinical use of aggressive therapeutic methods such as surgery, radiation and chemotherapy to eliminate cancerous cells regardless of the inflicted extensive collateral damage. However, the effectiveness of traditional anti-cancer strategies is increasingly challenged by treatment failures such as interpatient responsiveness, onset of chemoresistance, and local and distal recurrence, which are partly attributable to the genetic heterogeneity and genome instability of tumors and continuous tumor evolution [137]. Tumor evolution follows a Darwinian model, which also predicts the insufficiency of targeting the cancer epithelium alone, underscoring the need for alternative therapeutic strategies.

**Fig. 2:**
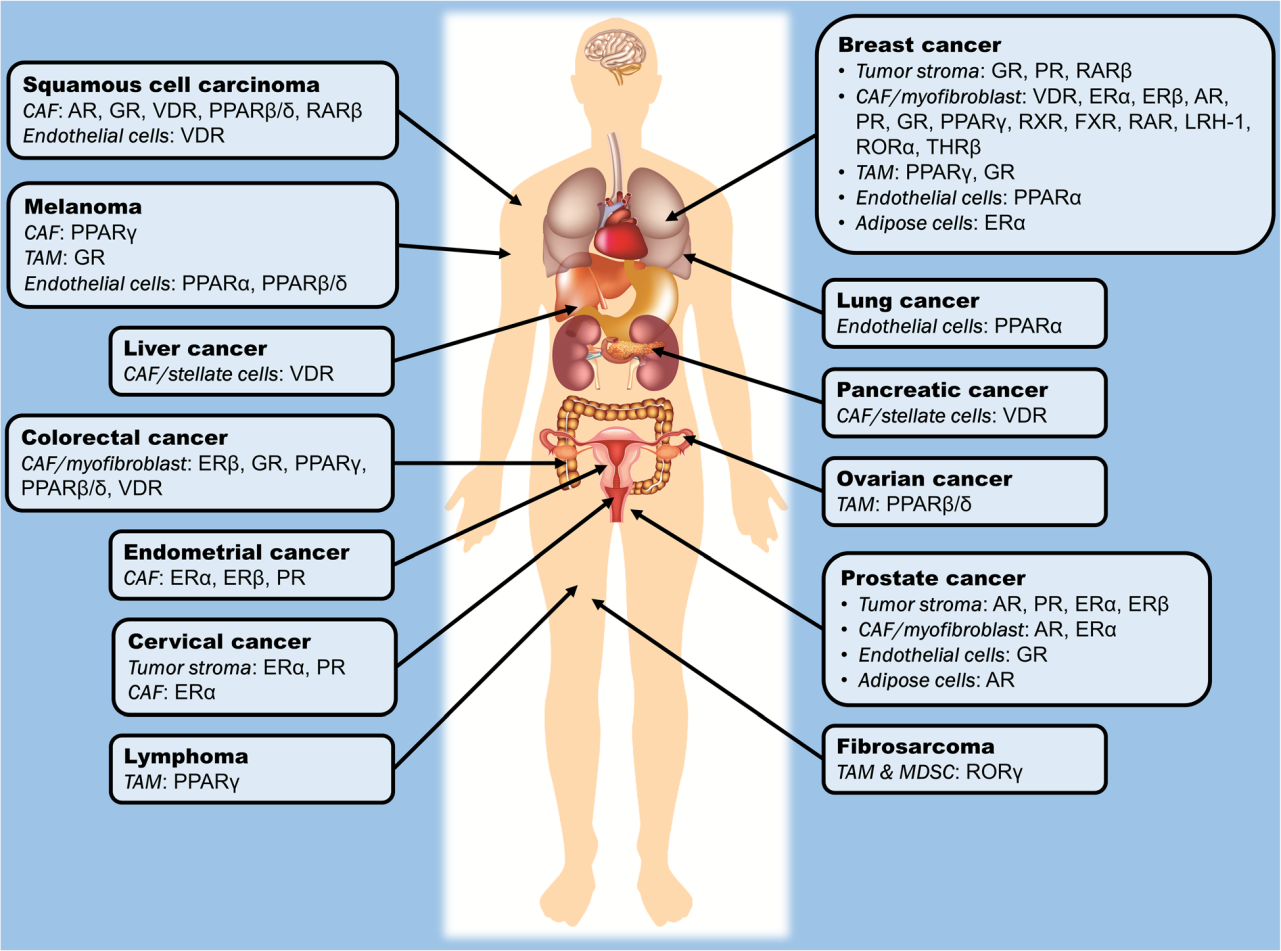
Summary of the so far identified nuclear receptors in the tumor microenvironment which play an active role in the modulation of oncogenic processes in different cancer types. AR, androgen receptor; CAF, cancer-associated fibroblast; ER, estrogen receptor; FXR, farnesoid X receptor; GR, glucocorticoid receptor; LHR-1, liver homolog receptor-1; MDSC, myeloid-derived suppressive cells; PPAR, peroxisome proliferator-activated receptor; PR, progesterone receptor; RAR, retinoic acid receptor; ROR, RAR-related orphan receptor; RXR, retinoic X receptor; TAM, tumor-associated macrophage; THR, thyroid hormone receptor; VDR, vitamin D receptor

Stroma-directed anticancer therapy will require a different therapeutic approach aimed at multiple and interacting cells. Stromal cells are generally considered to be more genetically stable, and thus the occurrence of mutations that may lead to resistance to drug treatments are minimal compared with the large tumor mutation burden observed in cancer cells. By consolidating the NR profile of various stromal cells across different tumor types, we can highlight NRs that have been thus far identified to regulate the assistive properties of tumor stroma in carcinogenesis, as summarized in Table 1 and Fig. 2. Certain NRs are clearly consistently observed across different tumor types; for instance, VDR, PPARs, ER, GR and AR in CAFs, as well as GR and PPARs in TAMs and endothelial cells. Modulating the activities of these NRs in stromal cells may potentially serve as a common adjunct therapy for the treatment of a wide range of cancers. In this context, by targeting NRs in stromal cells, the resultant physiological changes and drug responses could be more predictable, explaining why selected NRs, notably PPARs and GR, are consistently found to be crucial modulators of tumorigenesis in a cancer type-independent manner.

**Table 1:** Summary of existing research studies that exploited NRs in different tumor stromal cells and investigated the impacts on carcinogenesis and tumor microenvironment

Stromal cell types	Cancer types	Models	Target NR(s)	Agonists/antagonists	Key findings	References
CAF	Cutaneous squamous cell carcinoma (SCC)	Cell culture; Mice with SCC + CAF xenograft	48 known NRs in cell-based studies; AR and RARβ in mice models	Transfection of siRNA/expression vectors of targeted NRs into CAFs; PPARβ/δ agonist – GW0742; VDR agonist – EB1089; GR agonist – Fluticasone propionate; RARβ antagonist – LE135; AR antagonist – Bicalutamide	• PPARβ/δ, VDR, GR, RARβ and AR in CAFs are important modifiers of tumorigenic activities. • Concurrent therapy of cisplatin, LE135 and bicalutamide attenuated chemoresistance in mice tumor xenografts.	[15]
CAF	Prostate cancer	Cell culture; Mice with prostate cancer (PC3) + CAF xenograft	AR	Transfection of AR-expressing vectors into CAFs	• *AR*-expressing stromal cells suppressed prostate cancer growth and invasiveness in vitro and in vivo.	[20]
CAF	Prostate cancer	Cell culture; Mice with prostate cancer (PC3) + CAF xenograft	AR	Transfection of AR-expressing vectors into CAFs	• Low stromal *AR* expression decreased castration-induced apoptosis. • Loss of AR signaling in CAFs disrupted extracellular matrix integrity, promoting cancer cells invasion.	[22]
CAF	Prostate cancer	Cell culture	AR	Transfection of siRNA into CAFs	• Knockdown of *AR* in CAFs downregulated the expression of various growth factors and impaired the growth of tumor cells.	[28]
CAF	Prostate cancer	Cell culture	AR	Agonist – R1881; Antagonist – RD162	• Migration of prostate cancer cells was inhibited by conditioned medium of CAFs treated with R1881, but was reversed by RD162.	[29]
CAF	Prostate cancer	Cell culture	AR	Knockdown with AR antisense oligonucleotides	• Suppression of *AR* expression in CAFs inhibited cancer cell growth, but promoted stem cell phenotypes.	[30]
CAF	Breast cancer	Cell culture	AR	Agonist – Mibolerone	• Exposure of conditioned medium from Mibolerone-treated CAFs reduced breast cancer cell motility.	[31]
CAF	Prostate cancer	Cell culture; Mice with prostate cancer (22RV1) + CAF xenograft	ERα	Transfection of ERα-expressing vectors into CAFs	• Conditioned medium from *ERα*-expressing CAFs stimulated proliferation of various prostate cancer cell lines. • Co-implantation of *ERα*-expressing CAFs and prostate cancer cells increased tumor size in mice.	[36]
CAF	Prostate cancer	Cell culture; Mice with prostate cancer (22RV1) + CAF xenograft	ERα	Transfection of ERα-expressing vectors into CAFs	• Stromal ERα reduced cancer cell invasion. • Mice co-injected with *ERα*-expressing CAFs and prostate cancer cells had less tumor foci, less metastases and reduced angiogenesis.	[37]
CAF	Prostate cancer	Cell culture; Mice with prostate cancer (22RV1) + CAF xenograft	ERα	Transfection of ERα-expressing vectors into CAFs	• *ERα*-expressing CAFs suppressed cancer invasiveness via reduced macrophage infiltration.	[38]
CAF	Breast cancer	Cell culture	ERα, PR	Using CAFs isolated from ERα^+^/PR^+^ or ERα^−^/PR^−^ breast tumors	• Cancer cells co-cultured with ERα^+^/PR^+^ tumor-derived CAFs had higher tamoxifen sensitivity.	[39]
CAF	Cervical cancer	Cell culture	ERα	Agonist – Estradiol; Antagonist – ICI 182780, methyl piperidino pyrazole	• ERα antagonists downregulated genes associated with cell cycle, metabolism and angiogenic processes.	[40]
CAF	Prostate cancer	Cell culture	PR	Transfection of PRα- and PRβ-expressing vectors into CAFs	• Conditioned medium from *PR*-expressing CAFs inhibited cancer cell migration and invasiveness, but not cell proliferation.	[51]
CAF	Prostate cancer	Cell culture	PR	Transfection of PRα- and PRβ-expressing vectors into CAFs	• PR regulated prostate stromal cell differentiation.	[52]
CAF	Colorectal cancer	Cell culture	GR	Agonist – Dexamethasone	• Dexamethasone induced GR translocation into CAF nucleus, negatively regulating the expression of pro-inflammatory genes and paracrine factors that promote cancer invasiveness.	[56]
CAF	Colorectal cancer	Cell culture	GR	Agonist – Dexamethasone	• Conditioned medium from dexamethasone-treated CAFs decreased cancer cell proliferation and invasiveness.	[57]
CAF	Colorectal cancer	Cell culture	GR	Agonist – Dexamethasone	• Conditioned medium from dexamethasone-treated CAFs impaired endothelial cell migration by altering CAF secretome.	[58]
CAF	Pancreatic cancer	Cell culture; Mice with pancreatic ductal adenocarcinoma (PDA) xenograft	VDR	Agonist – Calcipotriol	• Calcipotriol maintained the quiescent state of pancreatic stellate cells. • Co-administration of calcipotriol and gemcitabine decreased tumor volume, enhanced intratumoral gemcitabine and increased survival rates of mice with tumor xenograft.	[62]
CAF	Liver cancer	Cell culture; p62KO mice	VDR	Agonist – Calcipotriol	• p62 is a mediator of calcipotriol-induced VDR activation which prevented hepatic stellate cell activation.	[64]
CAF	Pancreatic cancer	Cell culture	VDR	Agonist – Calcitriol	• Calcitriol modified miRNA composition in CAF exosomes.	[65]
CAF	Breast cancer	Cell culture	PPARγ, RXR	PPARγ agonist – Pioglitazone; RXR agonist – 6-OH-11-O-hydrophenanthrene	• PPARγ/RXR agonists inhibited NF-κB and metalloproteinase activities in CAFs.	[70]
CAF	Melanoma	Cell culture	PPARγ	PPARγ agonist – Ciglitazone, troglitazone, WY14643, 15d-PGJ2	• 15d-PGJ2 inhibited the growth of CAFs and tube formation of endothelial cells.	[71]
CAF	Colorectal cancer	Cell culture; Fibroblast-specific PPARβ/δ knockout mice	PPARβ/δ	*PPARβ/δ* gene knockout	• Fibroblast-specific *PPARβ/δ* knockout mice had prolonged survival and fewer intestinal polyps. • CAFs with *PPARβ/δ* deletion reduced oxidative stress in cancer epithelium.	[72]
CAF	Breast cancer	Cell culture; Mice with breast cancer (MCF-7) + CAF xenograft	FXR	Agonist – GW4064	• Conditioned medium from GW4064-treated CAFs inhibited leptin signaling, growth, motility and invasiveness of cancer cells. • GW4064-treated tumors had smaller sizes in in vivo xenograft studies.	[77]
CAF	Breast cancer	Cell culture	FXR	Agonist – GW4064	• GW4064 reduced migration and contractility of CAFs besides inhibiting growth and motility of cancer cells.	[78]
CAF	Breast cancer	RARβ knockout mice	RARβ	*RARβ* gene knockout	• *RARβ* knockout mice had reduced angiogenesis, inflammatory cell infiltration and myofibroblast count.	[79]
TAM	–	Cell culture	GR	Agonist – Dexamethasone	• Dexamethasone-dependent GR activation promoted alternative differentiation of monocytes to macrophages with a M2 phenotype.	[83]
TAM	Breast cancer	Mice with breast cancer (TS/A) + TAM xenograft	GR	Agonist – Glucocorticoid	• TAMs exposed to a mixture containing conditioned medium from *MS4A8A*-expressing tumors, interleukin-4 and glucocorticoids enhanced tumor growth in mice with tumor xenograft.	[84]
TAM	–	Inflammatory cell-specific ERα and ERβ knockout mice	ERα, ERβ	*ERα* and *ERβ* gene knockout; ER agonist – 17β-estradiol	• ERα signaling promoted alternative activation of macrophages.	[90]
TAM	Breast cancer	Cell culture; Female MMTV-PyMT mice	PPARγ	–	• Cleavage of PPARγ by caspase-1 promoted TAM differentiation. • Inhibition of caspase-1 attenuated caspase-1/PPARγ interaction and suppressed tumor growth.	[98]
TAM	Ovarian cancer	Cell culture	PPARβ/δ	Agonist – L165041; Inverse agonist – ST247, PT-S264	• Activation of PPARβ/δ upregulated immunity- and tumorigenesis-related genes in TAMs. • Inverse agonists of PPARβ/δ reversed the abnormal gene expression.	[99]
TAM	–	Cell culture	PPARγ	Agonist – Rosiglitazone, 15d-PGJ_2_	• PPARγ agonists reversed the suppressive effect of TAMs on antitumor cytotoxic T-cells.	[100]
TAM	Breast cancer	Cell culture; Macrophage PPARγ knockout mice	PPARγ	Agonist – Rosiglitazone	• Macrophage *PPARγ* ablation promoted breast tumor growth and nullified anti-tumor effects of rosiglitazone.	[101]
TAM & MDSC	–	Mice with fibrosarcoma (MN/MCA1) xenograft; MMTV-PyMT mice	RORγ	*RORC1* gene knockout	• RORγ protected MDSCs from apoptosis, promoted TAM differentiation and prevented neutrophil infiltration into tumor, thus leading to tumor growth and metastasis.	[107]
Endothelial cell	–	Cell culture; Tie2CrePPARγ^flox/flox^ mice	PPARγ	*PPARγ* gene knockout	• Deletion of *PPARγ* impaired angiogenesis and cellular migration in vitro and in vivo.	[114]
Endothelial cell	Melanoma, lung cancer, glioblastoma, fibrosarcoma	Cell culture; Mice with melanoma (B16-F10), Lewis lung carcinoma, glioblastoma (U87) or fibrosarcoma (HT1080) xenograft	PPARα	*PPARα* gene knockout; Agonist – Fenofibrate, gemfibrozil, bezafibrate, WY14643 and 5, 8, 11, 14-eicosatetraynoic acid	• Fenofibrate strongly suppressed endothelial cell proliferation, angiogenesis and primary tumor growth in mice. • Anti-angiogenic effect of fenofibrate was reversed by *PPARα* knockout.	[115]
Endothelial cell	–	Cell culture; C57BL6 mice	PPARβ/δ	Agonist – GW501516	• GW501516 induced endothelial cell proliferation and angiogenesis in vitro and in vivo.	[116]
Endothelial cell	Lung cancer	PPARβ/δ knockout mice	PPARβ/δ	*PPARβ/δ* gene knockout	• *PPARβ/δ* knockout impaired endothelial cell maturation, causing diminished blood flow to the tumors and abnormal microvascular structures.	[117]
Endothelial cell	Squamous cell carcinoma	Cell culture	VDR	Agonist – Calcitriol	• Tumor-derived endothelial cells were sensitive to the anti-proliferative effects of calcitriol.	[119]
Endothelial cell	Squamous cell carcinoma	Cell culture	VDR	Agonist – Calcitriol	• Calcitriol induced cell cycle arrest and apoptosis in tumor-derived endothelial cells, which were attributable to CYP24 inhibition.	[121]
Endothelial cell	Squamous cell carcinoma	Cell culture	VDR	Agonist – Calcitriol	• Methylation silencing of CYP24 promoter led to differential sensitivity to calcitriol-dependent growth inhibition in endothelial cells.	[123]
Endothelial cell	–	Cell culture	GR	Agonist – Dexamethasone, cortisol; Antagonist – RU38486	• GR agonists blocked microvessel tubule formation, but did not affect viability. The anti-angiogenic effects were reversed by RU38486.	[128]
Endothelial cell	Melanoma	Cell culture; Mice with melanoma (B16.F10) xenograft	GR	Agonist – Prednisolone, dexamethasome, budesonide, methylprednisolone	• All GR agonists inhibited tumor size and growth of endothelial cells.	[129]
Extra-hematopoietic Tie2-positive cells	Melanoma, lung cancer, breast cancer	Ovariectomized mice with melanoma (B16K1), Lewis lung carcinoma (LL2) or breast cancer (4 T1) xenograft	ERα	Agonist – Estradiol	• Extra-hematopoietic Tie2-expressing cells were responsible for increased tumor growth and intratumoral vessel density induced by estradiol treatment.	[130]
Adipocyte	Prostate cancer	Cell culture; Mice with prostate cancer (22RV1) xenograft	AR	–	• Recruitment of adipocytes to prostate cancer cells enhanced cancer invasiveness via suppression of AR activity and induction of TGF-β1/Smad/MMP9 signals.	[135]
Adipocyte	Breast cancer	Cell culture	ERα	–	• Adipocytes exposed to hypoxic condition triggered ERα suppression and promoted endothelial-to-mesenchymal transition of breast cancer cells.	[136]

For stroma-directed therapy to be a viable strategy as part of a multimodality approach or as adjunctive treatment to conventional tumor treatment, we also need to address the relative population of different stromal cells in different tumor types. For example, CAFs are relatively rare in brain, renal and ovarian cancers. In such instances, the depletion of CAFs or the disruption of CAF functions is likely to provide only marginal benefits. Similarly, while next-generation cancer treatment using immunotherapies such as PD-1 checkpoint blockade and Chimeric Antigen Receptor T-cell (CART) therapy are swiftly gaining attention, the efficacy of CAR-T therapy is dependent on the immune cell interactions in the TME [138, 139]. A recent characterization of immune infiltrates has shown that tumor genotypes, such as the tumor mutation burden, determine immunophenotypes and tumor escape mechanisms [140]. In cases where immunotherapy is less successful, stroma-directed therapy targeting other stromal cells may rise to be the predominant player. Moreover, if the efficacy and universality of stroma-directed therapy by targeting NRs are validated, the strategy can even be used to treat rare cancers simply because of the comparable physiological functionality of stromal cells in TME. These speculations and effectiveness of NR-based stroma-directed therapy can be further tested by extensive exploration of the NR signatures in TME across different types of cancers.

### Limitations, challenges, and future perspectives

To a certain extent, manipulating NRs of key tumor-supporting cells can sensitize tumor cells to anti-cancer treatments by interfering with the stroma-tumor crosstalk. However, current knowledge is still too incomplete for reliable translation into favorable clinical outcomes for different cancer types because of several limitations. First, the available data are derived primarily from hormone-dependent tumors, most notably breast and prostate cancers. Hence, our understanding of the roles of NRs in TME is fundamentally based on cancer-associated cells that are more actively involved in steroid hormone modulation and signaling. The effects of steroids differ from cancer to cancer [141], raising concerns about the generalizability of the results to cancers that are less hormone-dependent. Second, concerning the abovementioned limitation, current findings mostly include studies of steroid hormone NRs such as GR, ER, AR, and PR, because the development of hormone-dependent cancers is highly sensitive to steroids, facilitating detection of the biological roles of steroid receptors in tumorigenesis. As a result, our knowledge about NRs in TME is markedly skewed towards steroid receptors. In contrast, orphan NRs such as ERRs, RORs and LRH-1 have demonstrated a strong linkage with carcinogenesis [142]. However, exploiting them as a potential cancer therapy is underappreciated due to the lack of well-characterized ligands. This situation is anticipated to change in the near future because the US Food and Drug Administration has recently approved the first use of RNA-based gene silencing drug (siRNA) to knock down the expression of defective transthyretin for the treatment of polyneuropathy in patients with hereditary transthyretin-mediated amyloidosis [143, 144]. Given that targeting orphan NRs with RNA interference technology could someday become a therapeutic option, the recent approval is believed to have sparked more intensive research on the impacts of orphan NR suppression in cancer development.

Furthermore, the roles of NRs in TME have been established mainly based on cell culture studies via coculturing methods or with the use of conditioned medium from tumor-supporting cells. Empirical data from in vivo animal studies of TME are limited because cell-specific activation or inhibition of an NR, especially with a pharmacological approach, is remarkably challenging in animal models. Although genetic engineering can be used to obtain targeted stimulation or knockdown in animals [36, 37], it is associated with tedious preparation, relatively high costs and arduous administration, rendering this approach less desirable in actual clinical settings compared with the use small molecules. However, cell-specific modulation of the specifically targeted NR is crucial because the same receptor can have opposing effects in different cancer-associated cells. This phenomenon is demonstrated by GR, the activation of which in cancer-associated myofibroblasts reduces tumor proliferation [57] but promotes the M2 phenotype in macrophages, thus contributing to TAM differentiation and consequently tumor promotion [83]. Hence, given the heterogeneity of cellular populations in TME and their diverse physiological response to NR modulation, future research should also focus on the development of cell-specific drug delivery to achieve targeted manipulation of NR signaling in relevant cells.

The effects of NRs in TME on exosomes have scarcely been explored. Considering the vital roles of exosomes in cell-cell communication, which mediates various oncogenic processes, it is worthwhile to investigate how NR signaling in cancer-supporting cells calibrates the stroma-tumor interaction by regulating the exosomal content and liberation. Additionally, stroma-tumor communication is a dynamic and reciprocal action. Therefore, understanding how neighboring cancer cells affect NR signaling in the cancer-associated cells and downstream functional alterations can further reveal the true nature of TME. Essentially, in-depth dissection of the interplay between tumor-supporting cells and malignant cells may reveal additional exploitable targets to improve cancer therapy.

## Conclusions

NRs of tumor-supporting cells in TME play an essential role in various oncogenic processes. The NR signature of TME can serve as a crucial marker to pinpoint the fragility of the disease and guide the therapeutic strategy, with the ultimate goal of improving cancer prognosis. In light of the striking druggability of NRs, the future clinical prospect of developing a TME-oriented cancer therapy by targeting these receptors is promising. Among the 48 NRs in humans, the oncogenic functions of VDR, PPARs, AR, ER and GR in tumor-supporting cells are the best-characterized to date. Evidence of other receptors, such as RARβ, RORγ, and FXR, is limited yet promising. Given the heterogeneity of cellular populations within TME, more intensive research in understanding the molecular mechanisms of cell-cell interactions and how to master intercellular communication is of paramount importance. The ability to exploit NRs in TME in a highly specific and precise manner, in this case, can lay the foundation for precision medicine in cancer therapy and may even allow us to transform tumor-supporting cells into tumor foes.

## Abbreviations

AR: Androgen receptor
CAF: Cancer-associated fibroblast
ECM: Extracellular matrix
ER: Estrogen receptor
FXR: Farnesoid X receptor
GR: Glucocorticoid receptor
IL: Interleukin
LRH-1: Liver receptor homolog-1
MDSC: Myeloid-derived suppressor cells
NR: Nuclear receptor
PGC-1β: PPARγ coactivator 1β
PPAR: Peroxisome proliferator-activated receptor
PR: Progesterone receptor
RAR: Retinoic acid receptor
ROR: RAR-related orphan receptor
RXR: Retinoic X receptor
TAM: Tumor-associated macrophage
TGFβ: Transforming growth factor β
TME: Tumor microenvironment
TNFa: Tumor necrosis factor α
VDR: Vitamin D receptor
VEGF: Vascular endothelial growth factor
